# Store-Operated Calcium Entry Inhibition and Plasma Membrane Calcium Pump Upregulation Contribute to the Maintenance of Resting Cytosolic Calcium Concentration in A1-like Astrocytes

**DOI:** 10.3390/molecules28145363

**Published:** 2023-07-12

**Authors:** Joana Poejo, María Berrocal, Lucía Saez, Carlos Gutierrez-Merino, Ana M. Mata

**Affiliations:** 1 Instituto de Biomarcadores de Patologías Moleculares (IBPM), Universidad de Extremadura, 06006 Badajoz, Spain; joanapoejo86@gmail.com (J.P.); mabeca@unex.es (M.B.); 2 Departamento de Bioquímica y Biología Molecular y Genética, Facultad de Ciencias, Universidad de Extremadura, 06006 Badajoz, Spain; lucia.sm.9682@gmail.com

**Keywords:** A1 astrocytes, complement component C3, amyloid β, cytosolic calcium, intracellular calcium homeostasis, store-operated calcium entry, plasma membrane calcium pump

## Abstract

Highly neurotoxic A1-reactive astrocytes have been associated with several human neurodegenerative diseases. Complement protein C3 expression is strongly upregulated in A1 astrocytes, and this protein has been shown to be a specific biomarker of these astrocytes. Several cytokines released in neurodegenerative diseases have been shown to upregulate the production of amyloid β protein precursor (APP) and neurotoxic amyloid β (Aβ) peptides in reactive astrocytes. Also, aberrant Ca^2+^ signals have been proposed as a hallmark of astrocyte functional remodeling in Alzheimer’s disease mouse models. In this work, we induced the generation of A1-like reactive astrocytes after the co-treatment of U251 human astroglioma cells with a cocktail of the cytokines TNF-α, IL1-α and C1q. These A1-like astrocytes show increased production of APP and Aβ peptides compared to untreated U251 cells. Additionally, A1-like astrocytes show a (75 ± 10)% decrease in the Ca^2+^ stored in the endoplasmic reticulum (ER), (85 ± 10)% attenuation of Ca^2+^ entry after complete Ca^2+^ depletion of the ER, and three-fold upregulation of plasma membrane calcium pump expression, with respect to non-treated Control astrocytes. These altered intracellular Ca^2+^ dynamics allow A1-like astrocytes to efficiently counterbalance the enhanced release of Ca^2+^ from the ER, preventing a rise in the resting cytosolic Ca^2+^ concentration.

## 1. Introduction

Neuroinflammatory cytokines secreted by activated microglia in the brain can induce the generation of A1-reactive astrocytes that are highly neurotoxic [1,2,3]. Also, it has been reported that neurotoxic A1 astrocytes are abundant in post-mortem brain samples of the neurodegenerative diseases most prevalent in humans, like Alzheimer’s and Parkinson’s diseases, as well as in Huntington’s disease, amyotrophic lateral sclerosis and multiple sclerosis [3]. Three cytokines secreted by activated microglia, interleukin-1α (IL-1α), tumor necrosis factor-α (TNF-α) and complement component 1q (C1q), acting together, are necessary and sufficient to induce the generation of highly neurotoxic A1 astrocytes [3]. Since astrocytes are the most abundant brain cells and are required for neuronal survival and for the maintenance of blood–brain barrier integrity [4], the production of A1 astrocytes is a harmful threat in brain degeneration. Indeed, astrocytes are increasingly viewed as critical contributors to neurological disorders [5]. A1 astrocytes can secrete pro-inflammatory mediators that induce neuroinflammation [3,5,6,7], which eventually lead to blood–brain barrier integrity breakdown and brain edema formation. Also, it has been proposed that A1 astrocytes secrete a yet-unknown neurotoxin that potentiates neuronal death [6]. Moreover, in a previous work, we showed that the induction of A1 astrocytes in rat brains through the intraperitoneal administration of 3-nitropropionic acid (NPA), an animal model of Huntington’s disease, precedes the brain damage leading to motor neurological dysfunction in NPA-induced neurodegeneration [8]. The complement component C3 has been widely used as a specific marker of A1 astrocyte generation [3,8,9], because C3 expression is strongly upregulated in A1-reactive astrocytes.

In addition, it has been shown that reactive astrocytes induced via 2-chloroethanol poisoning can stimulate microglia polarization and microglia activation [10], leading to a feed-forward cycle that is harmful to specific brain structures. Furthermore, reactive astrocytes induced by cytokines such as interleukin-1β, TNF-α and interferon-γ have been shown to upregulate the production of amyloid β protein precursor (APP) and β-site APP-cleaving enzyme 1 (BACE1), and secrete neurotoxic amyloid β (Aβ) peptides [11,12,13,14]. In our recent work with rats treated through the intraperitoneal administration of NPA, we noticed an enhanced production of neurotoxic Aβ peptides in the brain areas displaying the generation of A1 astrocytes, namely, the striatum and hippocampus, which are also the brain regions most prone to NPA-induced degeneration [9]. The challenging possibility of an enhanced production of neurotoxic Aβ peptides in A1 astrocytes cannot be reliably concluded from our results from the animal model, and deserves to be experimentally assessed in a cell culture model.

Although astrocytes are electrically non-excitable cells, intracellular calcium signaling plays a major role in the modulation of their activities [15,16], which is critical for the activity and normal functioning of proximal neurons in the brain. As noted by [16], spontaneous astrocyte calcium transients of similar magnitude and frequency have been shown in vitro, in situ and in vivo, and these events have been suggested to facilitate the synchronization of nearby neuronal activity [17,18]. In older mice with amyloid plaques, the proportion of astrocytes with calcium oscillations has been reported to be increased, along with the amplitudes of those oscillations [19]. Indeed, aberrant Ca^2+^ signals have been proposed as a hallmark of astrocyte functional remodeling in Alzheimer’s disease (AD) mouse models [19,20]. However, to the best of our knowledge, the putative changes in intracellular calcium homeostasis associated with the transformation of normal astrocytes into neurotoxic A1 astrocytes have not been reported elsewhere.

On the other hand, experimental evidence of intracellular calcium dysregulation by Aβ is now overwhelming (see [21] for a recent review). Aβ is a recognized hallmark of AD, and intraneuronal Aβ accumulation has been suggested to be an early pathological biomarker for the onset of AD [22]. Mutations in presenilins (PSENs), which contribute to over 90% of familial AD cases, can also modulate capacitative calcium entry, a refilling mechanism for depleted Ca^2+^ stores [23,24,25], and mutations in the PSEN2 gene enhance Ca^2+^ release from the endoplasmic reticulum (ER) through inositol trisphosphate receptors [26]. In turn, the increase in cytosolic calcium leads to calmodulin-mediated stimulation of the amyloidogenic protease BACE1 [27]. In addition, the treatment of neuronal and neuroblastoma cells with 1 μM soluble Aβ(1–42) increased BACE1 transcription, an effect that was reverted by an anti-Aβ(1–42) antibody [28]. Therefore, Aβ generates a positive feedback loop for Aβ production. Also, attenuated Ca^2+^ entry through store-operated calcium entry (SOCE) is consistently observed in sporadic AD and in skin fibroblasts from familial AD patients [29]. Recently, we showed that the Ca^2+^ entry through SOCE is highly sensitive to Aβ, because intracellular nanomolar concentrations of the Aβ oligomer can bind to stromal interaction molecule 1 (STIM1) and produce significant inhibition of Ca^2+^ entry [30]. On the other hand, the overexpression of plasma membrane calcium pumps (PMCAs) in striatal astrocytes has been shown to inhibit intracellular Ca^2+^ signals [31]. Interestingly, the modulation of PMCA activity has been suggested to be involved in the counterbalance of early cytosolic Ca^2+^ upregulation in primary cortical mouse astrocytes caused by Aβ [32]. These authors reported that the Aβ(25–35) fragment potentiated PMCA-mediated Ca^2+^ extrusion in Aβ-conditioned primary cultures of astrocytes, leading to the diminution of cytosolic calcium concentration. 

On these grounds, the aims of this work were to set up the experimental conditions of treatment with a cocktail of the cytokines IL-1α, TNFα and C1q to induce A1-like astrocytes in U251 cells in culture, in order to evaluate their capability to express APP and Aβ peptides, and to study the putative alterations of SOCE, resting cytosolic calcium concentration and PMCA expression levels in A1-like human astrocytic U251 cells compared to Control (untreated) cells.

## 2. Results

### 2.1. Selection of A1-like Astrocytes Using C3 Expression as Biomarker

**Figure 1:**
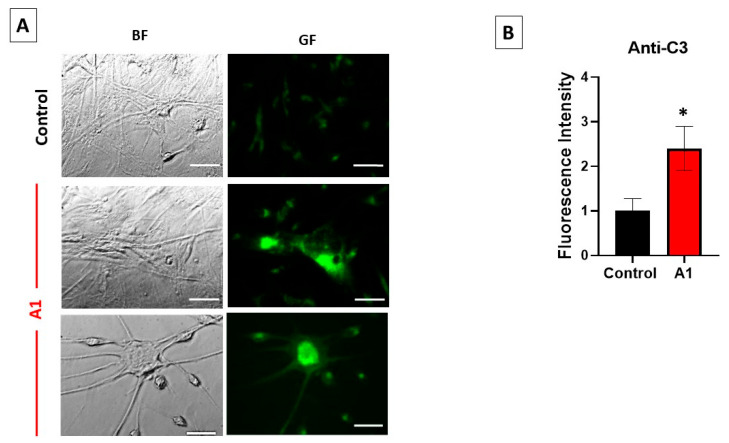
Enhanced expression of C3 in U251 astrocytes after treatment with the cytokines TNF-α (30 ng/mL), IL-1α (3 ng/mL) and C1q (400 ng/mL) for 24 h. (**A**) Representative fluorescence microscopy images of untreated U251 astrocytes (Control) and A1-like astrocytes induced by the treatment with a cocktail of cytokines stained with the anti-C3 antibody, as indicated in the Materials and Methods. All images were acquired with the same exposure time (0.3 s) using the Hamamatsu HCImage software of the fluorescence microscope. BF and GF stand for bright field and green fluorescence, respectively. The size of scale bars included in the microscopy images is 25 μm. (**B**) Mean ± SEM (standard error of the means) values of the fluorescence intensity of the soma of the Control (*n* = 21 cells) and A1-like astrocytes (*n* = 46 cells) stained with the anti-C3 antibody. Statistical analysis was carried out using the Student’s *t*-test. (*) *p* < 0.05 with respect to the Control.

After the treatment of U251 astrocytes with an inflammatory-mediator cocktail of cytokines, the larger cells with thick and large extensions display an expression of C3 several times higher than untreated Control U251 astrocytes, which are thinner and more elongated, and of much smaller size (see representative images in Figure 1). The analysis of the intensity of fluorescence in the images of cells stained with the anti-C3 antibody labeled with a secondary fluorescent antibody, acquired with the same exposure time (Figure 1), point out an increase in approximately 2.4 times (SEM ± 0.3) the expression level of C3 in the soma of A1-like astrocytes with respect to the non-transformed Control U251 astrocytes.

**Figure 2:**
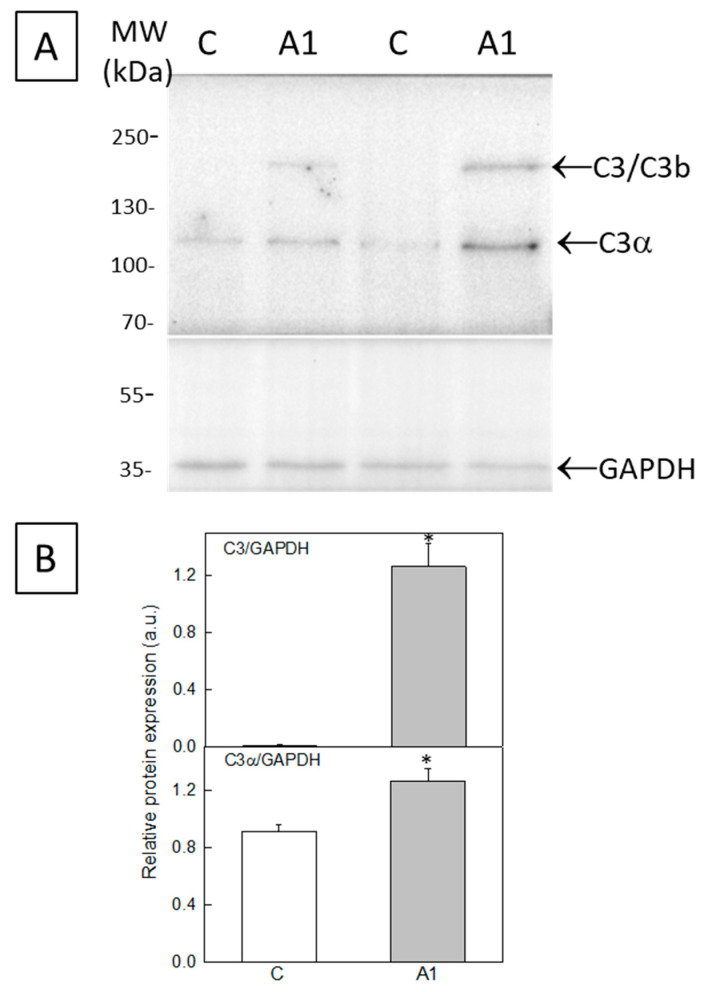
Expression of C3 in homogenates from astrocyte (U251 cell line) cultures. (**A**) Twenty-microgram homogenates from U251 cells untreated (C) or treated (A1-like) with TNF-α (30 ng/mL), IL-1α (3 ng/mL) and C1q (400 ng/mL) were loaded onto a 7.5–20% SDS-polyacrylamide gel, electrotransferred to a PVDF membrane, and immunostained with the anti-C3 antibody JF10-30. Representative blots from two different cultures are shown. The anti-GAPDH antibody was used as a protein loading Control. (**B**) The expression of C3 was quantified using Western blots from homogenates of four different Control and A1-like astrocytes cultures. Data were normalized to GAPDH and are represented as mean ± SEM values in arbitrary units. Statistical analysis was carried out using the Student’s *t*-test. (*) *p* < 0.05 with respect to each Control.

The enhanced expression of C3 in A1-like astrocytes was also experimentally assessed in the whole cell culture using Western blot. Figure 2A shows an increase in band intensities corresponding to C3b or C3 (~175–185 kDa) and to the C3α subunit (~116 kDa) in U251 astrocytes treated with cytokines (A1-like astrocytes), with respect to the untreated Control U251 astrocytes. The polyvinylidene difluoride (PVDF) membrane was cut below 70 kDa, and the lower part was used to quantify glyceraldehyde-3-phosphate dehydrogenase (GAPDH), which was selected as a marker to evaluate the protein load per lane. The quantitative analysis of band signal intensities relative to those of GAPDH shows a consistent increase in the expression of C3b or C3 and of the C3α subunit after the treatment of U251 astrocytes with cytokines (Figure 2B). Of note, the increase in C3/C3b expression is much higher than the increase in the C3α protein. Overall, the increase in the C3/C3b protein band is consistent with the large increase in immunoreactivity against the anti-C3 antibody observed via fluorescence microscopy in the giant cells with thick and large extensions after the treatment of U251 astrocytes with cytokines.

### 2.2. A1-like Astrocytes Express Higher Levels of Aβ Peptides Than Control U251 Astrocytes

Recently, we reported that an increase in A1 astrocytes during 3-nitropropionic acid-induced brain neurodegeneration was correlated with an increase in neurotoxic Aβ peptide production in the damaged brain areas [9]. Furthermore, several studies have shown that reactive astrocytes in culture can produce neurotoxic Aβ peptides [14,33,34]. On these grounds, we tested the possibility that U251-derived A1-like astrocytes also produce neurotoxic Aβ peptides.

**Figure 3:**
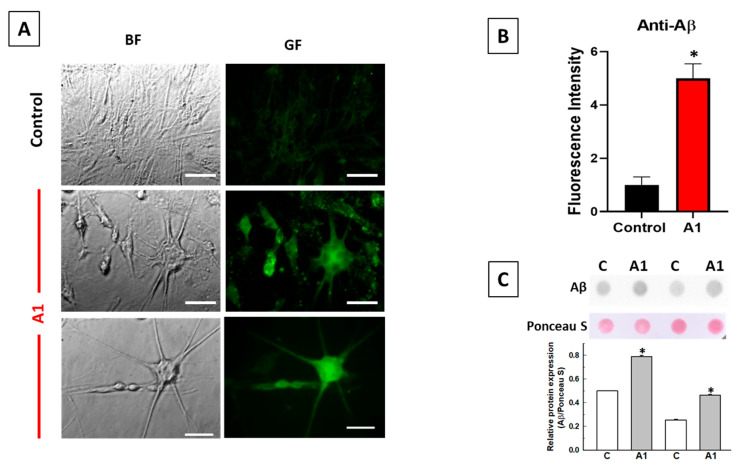
Enhanced expression of Aβ peptides in U251 astrocytes after treatment with the cytokines TNF-α (30 ng/mL), IL-1α (3 ng/mL) and C1q (400 ng/mL) for 24 h. (**A**) Representative fluorescence microscopy images of untreated U251 astrocytes (Control) and A1-like astrocytes induced by treatment with cytokines stained with the anti-Aβ antibody 6E10, as indicated in the Materials and Methods. All images were acquired with the same exposure time (0.3 s) using the Hamamatsu HCImage software of the fluorescence microscope. BF and GF stand for bright field and green fluorescence, respectively. The size of scale bars included in the microscopy images is 25 μm. (**B**) Mean ± SEM values of the fluorescence intensity of the soma of the Control (*n* = 19 cells) and A1-like astrocytes (*n* = 37 cells) stained with the anti-Aβ antibody. Statistical analysis was carried out using the Student’s *t*-test. (*) *p* < 0.05 with respect to the Control. (**C**) The expression of Aβ in homogenates from untreated (C) or treated (A1-like) U251 astrocytes was also quantified via dot blot, using 12 µg of homogenates and the anti-Aβ antibody, as described in the Materials and Methods. Representative dot blots from two different Control and A1-like astrocyte cultures are shown. Data were normalized to total protein stain using Ponceau S and are represented as mean ± SEM values in arbitrary units. (*) *p* < 0.05 with respect to each Control.

Figure 3 shows that the treatment of U251 astrocytes with cytokines elicits enhanced immunostaining, with the anti-Aβ antibody labeled with a secondary fluorescent antibody. In addition, immunostaining with the anti-Aβ antibody reveals a non-homogeneous intracellular distribution of Aβ toxic peptides and APP (Figure 3A), suggesting its association with intracellular organelles, as we previously reported in the HT-22 neuronal cell line [30]. The intensity of fluorescence images of large cells of A1-like astrocytes with thick extensions, which are those strongly stained with the anti-C3 antibody, is approximately 5 times (SEM ± 0.5) higher in A1-like astrocytes with respect to the non-transformed Control U251 astrocytes acquired with the same exposure time (Figure 3B). An analysis of Aβ expression was also carried out via dot blot (Figure 3C), using the same anti-Aβ antibody. As shown, the treatment of U251 astrocytes with cytokines significantly increased the expression of Aβ peptides with respect to the non-treated cells.

### 2.3. Store-Operated Calcium Entry (SOCE) Is Largely Decreased during the Transformation of U251 Astrocytes in A1-like Astrocytes 

**Figure 4:**
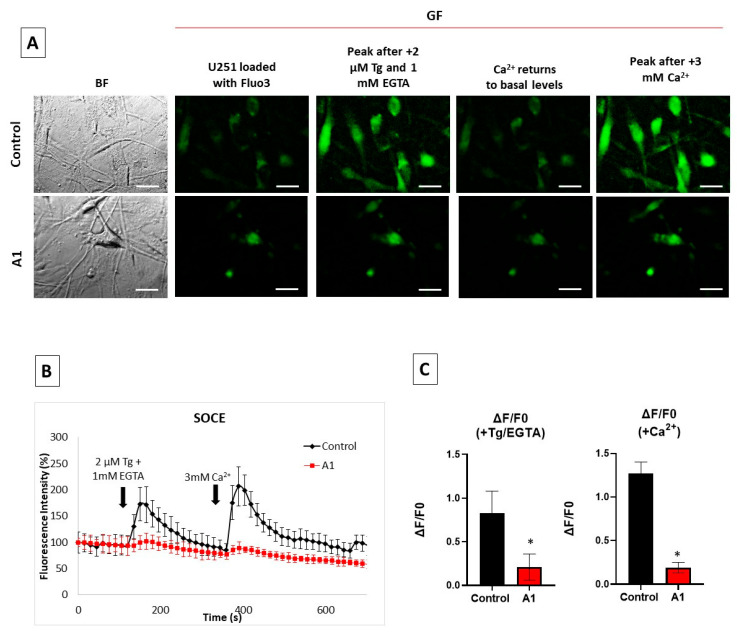
SOCE inhibition in U251 astrocytes after treatment with the cytokines TNF-α (30 ng/mL), IL-1α (3 ng/mL) and C1q (400 ng/mL) for 24 h. Untreated (Control) and treated U251 cells were loaded with 5 μM Fluo-3-pentaacetoxymethyl ester (Fluo3 AM) and 0.025% Pluronic^®^ F-127 for 1 h to experimentally evaluate Ca^2+^ imaging of SOCE. (**A**) Representative microscopy images of Fluo3-loaded untreated U251 astrocytes (Control) and cells treated with the cytokines (reactive A1-like astrocytes), acquired during SOCE experiments. The size of the scale bars included in the microscopy images is 25 μm. (**B**) Representative kinetic traces of untreated U251 astrocytes (black trace) and reactive A1-like astrocytes (red trace) after the addition of 2 μM Tg plus 1 mM ethyleneglycoltetraacetic acid (EGTA), indicated by the first arrow, for Ca^2+^ release from stores and after the addition of 3 mM Ca^2+^ to monitor Ca^2+^ entry through the plasma membrane (indicated by the second arrow). (**C**) Means of the average fluorescence intensity (ΔF/F0) relative to the Control, after the addition of Tg + EGTA (Ca^2+^ release from stores) or after the addition of Ca^2+^ (Ca^2+^ entry). Data are presented as the mean ± SEM values of the experiments, performed in triplicate with *n* = 30 Control U251 cells, and *n* = 9 reactive A1-like astrocytes. Statistical analysis was carried out using the Student’s *t*-test. (*) *p* < 0.05 with respect to each Control.

Neurotoxic Aβ peptides have been shown to impair intracellular calcium homeostasis in brain cells [21,35,36]. Moreover, several ER proteins have been shown to be involved in Aβ(1–42) production, and also in the Aβ(1–42) dysregulation of intracellular calcium, reviewed in [21]. In a recent work, we reported that nanomolar concentrations of the Aβ(1–42) peptide can inhibit SOCE [30]. Therefore, we experimentally measured the SOCE response in U251 astrocytes treated with cytokines and in untreated Control U251 astrocytes (Figure 4). 

We selected as A1-like astrocytes the cells displaying a higher expression of C3, monitored with the anti-C3 antibody, after the treatment with cytokines. Among them, we selected for intracellular Ca^2+^ measurements only larger cells with thick extensions that also had higher levels of Aβ, detected with the anti-Aβ(1–42) antibody, because they are the most reliable for the analysis of fluorescence intensity changes using the region of interest (ROI) tool of the HCImage software of our fluorescence microscope. Figure 4B shows that the peaks of the thapsigargin (Tg)-induced release of Ca^2+^ from the ER and the Ca^2+^ entry after depletion of the ER with Tg are largely decreased in these A1-like astrocytes with respect to the Control U251 astrocytes not treated with cytokines. The analysis of the results show a (75 ± 10)% decrease in Tg-induced Ca^2+^ release from the ER and (85 ± 10)% inhibition of Ca^2+^ entry after depletion of the ER with Tg (Figure 4C).

### 2.4. The Cytosolic Ca^2+^ Concentration Does Not Increase during the Transformation of U251 Astrocytes in A1-like Astrocytes, Which Express Higher Levels of PMCA Than the Control U251 Astrocytes

The above results show a decrease in Ca^2+^ stored in the ER in A1-like astrocytes. In addition, large A1-like astrocytes with thick extensions loaded with Fluo3 display higher-intensity fluorescence than the Control U251 astrocytes untreated with cytokines. Therefore, we measured cytosolic Ca^2+^ concentration to experimentally assess the possibility that the enhanced release of Ca^2+^ from the ER can lead to a sustained increase in cytosolic calcium concentration in A1-like astrocytes. The cytosolic Ca^2+^ concentration was measured as described in the Materials and Methods. Although Fluo3-loaded A1-like astrocytes display a higher intensity of fluorescence, our results point out that the A1-like astrocytes uptake Fluo3 more efficiently than the Control astrocytes not treated with cytokines, because the analysis of these results show cytosolic Ca^2+^ concentrations of 66 ± 19 nM and 82 ± 24 nM for the A1-like astrocytes (*n* = 8 cells) and for the Control astrocytes (*n* = 28 cells), respectively. These results were further confirmed using Fura2-loaded astrocytes.

**Figure 5:**
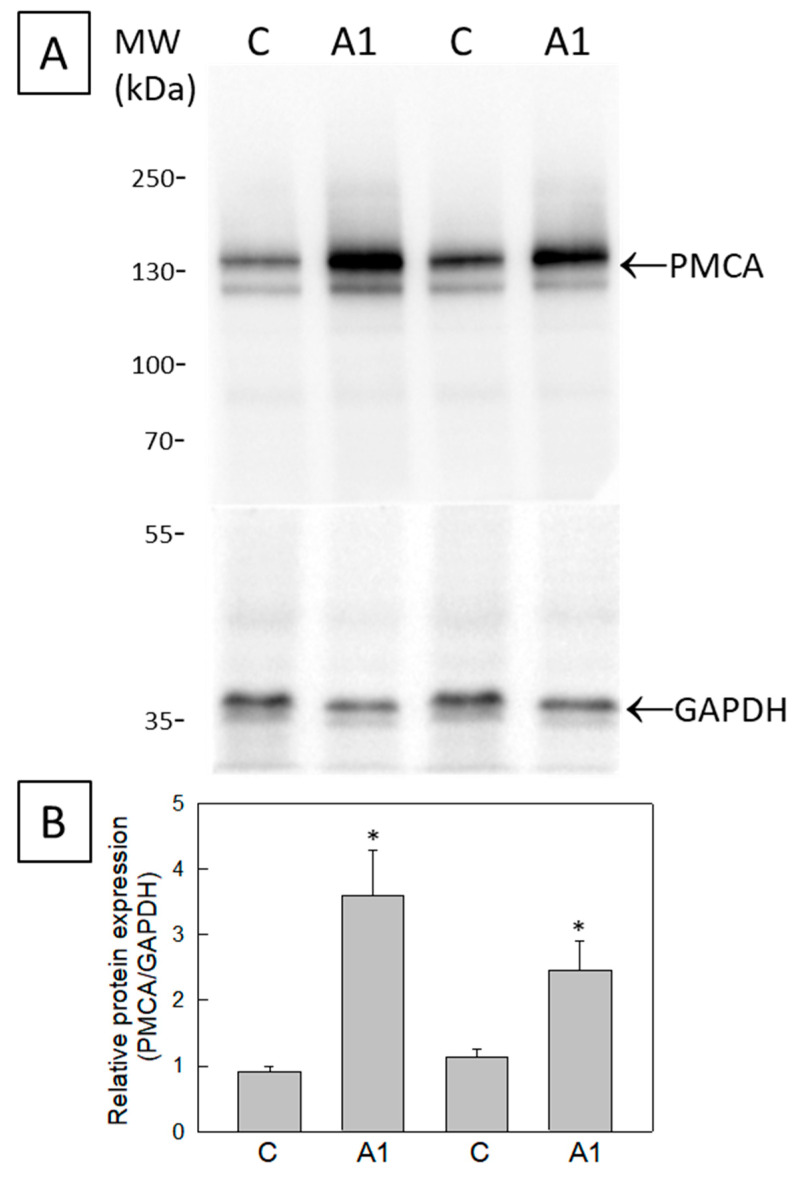
Expression of PMCA in homogenates from astrocyte (U251 cell line) cultures. (**A**) Twenty-microgram homogenates from U251 cells untreated (C) or treated (A1-like astrocytes) with TNF-α (30 ng/mL), IL-1α (3 ng/mL) and C1q (400 ng/mL), were loaded onto a 10% SDS-polyacrylamide gel, electrotransferred to a PVDF membrane, and immunostained with the PMCA antibody 5F10. The anti-GAPDH antibody was used as a protein loading control. (**B**) Quantification of PMCA protein levels in Control and A1-like astrocytes relative to GAPDH is shown as mean ± SEM values, in arbitrary units. Statistical analysis was carried out using the Student’s *t*-test. (*) *p* < 0.05 with respect to each Control.

Since PMCAs play a key role in Ca^2+^ extrusion and an increase in PMCA expression during development has been showed in astrocytes cultured in vitro [37], next, we measured the putative changes in the expression level of PMCAs induced by the treatment of U251 astrocytes with cytokines (Figure 5). The results show that PMCA is upregulated in A1-like astrocytes, which express around a three-fold (SEM ± 0.6) higher level of PMCAs relative to the Control U251 astrocytes not treated with cytokines.

## 3. Discussion

The results of this work point out that treatment with the cytokines TNF-α (30 ng/mL), IL-1α (3 ng/mL) and C1q (400 ng/mL) for 24 h produces a large increase in C3 or C3b expression in U251 cells, and also around a two-fold increase in C3α expression, which are biomarkers of reactive A1 astrocytes [3]. Thus, this treatment with cytokines is sufficient to elicit the significant generation of A1-like astrocytes. Fluorescence microscopy images of cells stained with the anti-C3 antibody show that the increase in C3/C3b/C3α expression is stronger in the soma of large cells displaying thick and long extensions. Therefore, we selected this cell phenotype to study the effect of the transformation of U251 cells in A1-like astrocytes on Aβ production and on resting cytosolic Ca^2+^ homeostasis. 

Many pro-inflammatory cytokines have been shown to upregulate APP in human neuroblastoma cells and non-neuronal cells such as human astrocyte cultures, as well as in the mouse brain [34]. For example, interleukin-1β has been shown to upregulate APP in human astrocytes and the U373MG human astrocytoma cell line [11,12]. Zhao et al. [14] have demonstrated that treatment for ≥48 h with cytokines including TNF-α and interferon-γ increase levels of endogenous BACE1 and APP, and stimulate amyloidogenic APP processing, in primary mouse astrocytes, leading to an increase in Aβ secretion. While there is little Aβ secretion from resting normal adult human astrocytes [38], a combination of interferon-γ and TNF-α has also been shown to induce Aβ secretion from primary human astrocytes and the U373 cell line [13]. In this work, we show that the treatment of U251 cells with the cytokines TNF-α (30 ng/mL), IL-1α (3 ng/mL) and C1q (400 ng/mL) for 24 h also produces a large increase in Aβ peptides immunoreactive with the anti-Aβ antibody in cells displaying an enhanced expression of C3/C3b/C3α, i.e., with the A1-like astrocyte phenotype. Of note, the anti-Aβ antibody 6E10 used in this work reacts to the toxic human Aβ peptides, such as Aβ(1–42) and Aβ(1–40), and also to their precursor forms, like APP, which contains the immunoreactive epitope against this antibody [39,40,41]. To the best of our knowledge, the production of toxic Aβ peptides by A1 astrocytes has not been reported elsewhere, and this is of particular relevance since A1 astrocytes are abundant in *post-mortem* brain samples of major human neurodegenerative diseases, including Alzheimer’s, Huntington’s and Parkinson’s diseases, as well as amyotrophic lateral sclerosis and multiple sclerosis [3]. Although under normal conditions, astrocytes are less likely to be significant generators of Aβ because neurons express higher levels of BACE1 than astrocytes [42,43], it should be noted that astrocytes outnumber neurons over five-fold in the brain [44,45]. Indeed, several studies have shown that reactive astrocytes can contribute to Aβ production in AD [13,34,46]. Moreover, Aβ produced by astrocytes may be more pathogenic than that produced by neurons. A large fraction of the Aβ species present in Aβ plaques are N-truncated [47,48,49], and it has been reported that the percentage of N-truncated Aβ secreted by astrocytes is much higher than that of Aβ secreted by neurons, with values of 60% and 20%, respectively [50]. As Aβ can stimulate astrocytes to secrete pro-inflammatory molecules and cytokines in vitro and in vivo [51,52,53], these results suggest that a feed-forward loop may operate during neurodegeneration mediated by A1 astrocytes. 

Aβ(1–42) and other neurotoxic β-amyloid peptides have been shown to alter intracellular Ca^2+^ homeostasis by acting on regulatory Ca^2+^-dependent proteins and Ca^2+^ transport systems, reviewed in [21]. Much experimental evidence supports that ER Ca^2+^ dysregulation can lead to Aβ(1–42) generation [21]. In this work, we found that the treatment of U251 cells with the cytokines TNF-α (30 ng/mL), IL-1α (3 ng/mL) and C1q (400 ng/mL) for 24 h produced a large depletion of ER calcium content, and also a large attenuation of SOCE. The latter result can be seen as a direct effect of Aβ(1–42) generation in these A1-like astrocytes, because we previously demonstrated that only nanomolar concentrations of intracellular Aβ(1–42) are needed to inhibit SOCE activity [30]. In addition, the enhanced release of calcium from the ER can generate a feed-forward cycle, leading to potentiation of the production of neurotoxic Aβ peptides in A1-like reactive astrocytes, since it has been demonstrated that Aβ-induced BACE1 upregulation can be blocked by preventing calcium influx through treatment with 2-aminoethoxydiphenyl borate, an inhibitor of inositol trisphosphate-dependent calcium release from the ER, and U73122, an inhibitor of phospholipase C [54]. 

The activity of astrocytes, the major glial cell-type in the mammalian brain [55], is largely dependent on intracellular calcium signaling [15]. Lee et al. [16] have reported that picomolar amounts of Aβ peptides can enhance spontaneous intracellular calcium transient signaling. Also, it has been shown that pathological Aβ can cause abnormal calcium influx and intracellular signaling in astrocyte cultures [56,57], and aberrant Ca^2+^ signals have been noted as a hallmark of astrocyte functional remodeling in AD mouse models [19,20]. However, in this work, we found that the resting cytosolic Ca^2+^ concentration does not increase in A1-like astrocytes with respect to the Control (untreated) U251 cells, despite the fact that ER calcium content is markedly decreased. On the contrary, the mean values obtained for the cytosolic Ca^2+^ concentrations are 20% lower in A1-like astrocytes than in the Control U251 cells. This result points out that the extrusion of cytosolic calcium operates more efficiently in the A1-like astrocytes than in the Control U251 cells. Indeed, our results show around a three-fold higher PMCA expression level in the A1-like astrocytes than in the Control U251 cells. Due to the major role of PMCAs in the cytosolic calcium extrusion of brain cells [58,59], the upregulation of PMCA activity is likely to efficiently counterbalance, in a short time, the impact of ER calcium release on the resting cytosolic calcium concentration of A1-like astrocytes, unveiling a defense mechanism against Aβ-induced abnormal and harmful calcium signaling in these cells. Interestingly, Pham et al. [32] have recently reported that Aβ(25–35) lowered the cytosolic calcium concentration in Aβ-preconditioned primary cortical mouse astrocytes through potentiated Ca^2+^ extrusion via PMCAs triggered by a rise in cyclic AMP. The molecular mechanisms involved in the upregulation of PMCAs in A1-like astrocytes are unclear at present, and its elucidation will need extensive experimental work that is out of the scope of this article.

In summary, we found that treatment of U251 cells with the cytokines TNF-α, IL-1α and C1q for 24 h elicits significant generation of A1-like astrocytes, which display increased production of APP and Aβ peptides. Compared with the Control U251 cells, these A1-like astrocytes show more than 50% depletion of the Ca^2+^ stored in the ER without an increase in cytosolic Ca^2+^ concentration, since the attenuation of Ca^2+^ entry through SOCE and the upregulation of PMCA expression can efficiently counterbalance the enhanced Ca^2+^ release from the ER in A1-like astrocytes. 

## 4. Materials and Methods

### 4.1. Materials

The human astroglioma U251 cell line was acquired from the American Type Culture Collection (Manassas, VA, USA). The primary antibody anti-C3 (JF10-30) and the fluorescent labeled secondary antibodies anti-rabbit IgG-Alexa488 (A11008) and anti-mouse IgG-Alexa488 (A11001) were from Invitrogen (Molecular Probes, Eugene, OR, USA). The primary antibody anti-Aβ (6E10) was purchased from Enzo Life Sciences (Farmingdale, NY, USA). The primary anti-PMCA antibody (clone 5F10) was purchased from Invitrogen. The primary anti-GAPDH antibody (GAPDH-0411) was obtained from Santa Cruz Biotechnology. IL-1α and TNF-α were supplied by Prepotech, and C1q was from BioRad. Fura-2 acetoxymethyl ester (Fura2 AM), Fluo3 AM, 4-Bromo-A23187 and Pluronic^®^F-127 were obtained from Biotium (Hayward, CA, USA). Thapsigargin (Tg) and ionomycin were purchased from Sigma-Aldrich. 

### 4.2. Cell Culture and Preparation of Reactive Astrocytes

U251 cells were cultured in high-glucose Dulbecco’s Modified Eagle’s Medium (DMEM) supplemented with 2 mM L-glutamine, 100 units/mL penicillin, 0.1 mg/mL streptomycin and 10% fetal bovine serum (FBS). For all experiments, U251 cells were seeded in 35 mm dishes at a density of 2.2 × 10^4^ cells/cm2 in the culture media described above and allowed to grow at 37 °C and 5% CO_2_. After 48 h, reactive astrocytes (named A1-like) were prepared by replacing the culture medium with FBS-free DMEM supplemented with glutamine and antibiotics. The next day, cells were stimulated for 24 h with a cocktail of the pro-inflammatory cytokines IL-1α (3 ng/mL), TNF-α (30 ng/mL) and C1q (400 ng/mL), as detailed by Liddelow et al. [3]. Non-reactive astrocyte cells (Control group) were always maintained in DMEM supplemented with FBS.

### 4.3. U251 Cell Staining with C3 and Aβ Antibodies

A1-like astrocytes and Control (untreated U251) cells were seeded in 35 mm plates as described above. On the day of the experiment, cells were washed with MLocke’s K5 buffer (4 mM NaHCO_3_, 10 mM Tricine, 5 mM glucose, 2.3 mM CaCl_2_, 1 mM MgCl_2_, 154 mM NaCl and 5 mM KCl, pH 7.4 at 37 °C) to remove the phenol red remaining in the plates. Afterward, cells were fixed with 2.5% paraformaldehyde, 3 mM MgCl_2_, 2 mM EGTA and 0.32 M sucrose in PBS (5 mM sodium phosphate, 137 mM NaCl and 27 mM KCl, pH 7). Fixed and permeabilized cells were blocked with 1% bovine serum albumin in PBS supplemented with 0.2% Triton X-100 (PBST) for 1 h at 37 °C and washed three times with PBS (washing step). Then, cells were incubated for 1 h at 37 °C with the respective target primary antibody in PBST: anti-C3 (dilution 1/200) or anti-Aβ (dilution 1/250 or 1/500). Thereafter, cells were washed and incubated for 1 h with the appropriate Alexa488-labeled secondary antibody in PBST (dilution 1/200) and rewashed. Green fluorescence (GF) images of Control and A1 cells were acquired with an excitation filter of 470 nm and a 510 nm dichroic mirror/520 nm emission filter, using the exposure times indicated for each case in the legends of the figures. Quantitative analysis of the average fluorescence intensity per pixel of selected neuronal soma was performed via HCImage software using the ROI tool, as in previous works [30,60,61,62].

### 4.4. Cell Homogenate Preparation, and Western Blot and Dot Blot Analyses

Cells were harvested, washed twice with PBS and centrifuged for 3 min at 1700 g to collect cell pellets. The Omni Tissue Master 125 with a 5 mm Probe was used to resuspend the pellet in 10 mM N-[2-hydroxyethyl] piperazine-N′-[2-ethanesulfonic acid] (HEPES)/KOH, at pH 7.4, with 0.32 M sucrose, 0.5 mM MgSO_4_, 0.1 mM phenylmethanesulfonyl fluoride, 2 mM 2-mercaptoethanol and a protease inhibitor cocktail solution (Roche Diagnostics). The homogenate was then centrifuged for 1 min at 5000 g, and the supernatant was collected and stored at −80 °C in small aliquots. The protein concentration was determined using the Bradford method [63].

Proteins from 20 µg homogenates were separated using 10% or 7.5–20% gradient polyacrylamide sodium dodecyl sulfate (SDS) gels [64] and transferred onto PVDF membranes. Immunodetection of C3, PMCA and GAPDH was performed by incubating the PVDF membranes with the specific primary antibodies anti-C3 (dilution 1/1000), anti-PMCA (dilution 1/1000) and anti-GAPDH (dilution 1/3000) overnight at 4 °C, followed by 1 h of incubation at room temperature with the appropriate peroxidase-conjugated anti-mouse or anti-rabbit secondary antibodies, and developed using an Enhanced Chemiluminescence (ECL) substrate. 

Immunodetection of total Aβ peptides was carried out via dot blot. Homogenates (12 µg) from U251 cells, untreated (C) or treated (A1-like astrocytes) with TNF-α (30 ng/mL), IL-1α (3 ng/mL) and C1q (400 ng/mL), were spotted onto a nitrocellulose membrane, sealed on a Bio-Dot^®^ SF (Bio-Rad, Hercules, CA, USA). Total protein was visualized via Ponceau S protein staining. After blocking and washing, the membrane was incubated with the anti-Aβ antibody (6E10, dilution 1/1000) overnight at 4 °C. The membrane was incubated with peroxidase-conjugated anti-mouse secondary antibody and developed using the Enhanced Chemiluminescence (ECL) substrate.

### 4.5. Intracellular Cytosolic Ca^2+^ Measurements

Intracellular Ca^2+^ concentration ([Ca^2+^]i) was measured in Control U251 cells and in A1-like astrocytes with the fluorescent probes Fluo3 AM and Fura2 AM, as previously described [30,60]. Briefly, cells were loaded with 5 μM of Fura2 AM or Fluo3 AM plus 0.025% Pluronic^®^ F-127 with continuous and gentle mixing for 1 h at 37 °C and 5% CO_2_. Afterward, cells were washed once with 1 mL of MLocke’s K5 buffer, and the 35 mm culture dishes were placed on the thermostatic plate (Warner Instrument Co., Hamden, CT, USA) of a Nikon Diaphot 300 inverted epifluorescence microscope (Tokyo, Japan) at 37 °C with an NCF Plan ELWD 40× objective. Images of Fluo3-loaded cells were acquired using a Hamamatsu Orca-R2 CCD camera (binning mode 2 × 2) with an excitation filter of 470 nm, and a dichroic mirror of 510 nm with an emission filter of 520 nm [30]. Images of Fura2-loaded cells were recorded using 340 and 380 nm excitation filters and a 510 nm dichroic mirror/520 nm emission filter [60,62]. 

For the determination of [Ca^2+^]i using the Fluo3 probe, 5 mM MnCl_2_ and 5 μg/mL of the nonfluorescent Ca^2+^ ionophore 4-Bromo-A23187 were added to Control U251 cells and to A1-like astrocytes, and the fluorescence microscopy images were recorded in kinetic mode to obtain the fluorescence intensity of Fluo3 saturated with Mn^2+^ [F(Mn-Fluo3)], which has been reported to be 20% of the value of fluorescence intensity upon saturating Ca^2+^ (Fmax) [65]. The fluorescence value of free Fluo3 (not bound to Ca^2+^), Fmin, is about 0.01·Fmax [65]. [Ca^2+^]i was measured using the formula: [Ca^2+^]i = K_d_ [(F − Fmin)/(Fmax − F), where F is the fluorescence intensity obtained with an excitation filter of 470 nm, and a 510 nm dichroic mirror/520 nm emission filter with a 0.1 s exposure time. The dissociation constant (K_d_) for the Fluo3/Ca^2+^ complex used to obtain [Ca^2+^]i was 390 nM, reported in the *Molecular Probes Handbook: A Guide to Fluorescent Probes and Labeling Technologies*, Tenth Edition (2005).

For the determination of [Ca^2+^]i using the Fura2 probe, [Ca^2+^]i was calculated using the following equation: [Ca^2+^]i = K_d_ × [(R − Rmin)/(Rmax − R)], where R is the measured fluorescence ratio (340/380), and Rmax and Rmin are the ratio values (340/380) for Ca^2+^-bound and Ca^2+^ -free Fura2-loaded cells. Rmax and Rmin were experimentally determined from steady-state fluorescence ratio (340/380) measurements after sequential addition to the culture medium of Fura2-loaded cells of (i) nonfluorescent Ca^2+^ ionophore 4-Bromo-A23187 (5 μg/mL) or ionomycin (5 μg/mL), and (ii) 10 mM EGTA, respectively. To obtain the values of [Ca^2+^]I, we used the reported dissociation constant (K_d_) of Fura2/Ca^2+^ of 224 nM [66].

The response of SOCE was determined in Fluo3-loaded Control U251 cells and in A1-like astrocytes. The depletion of the Ca^2+^ stores was triggered by adding 2 µM of the sarcoendoplasmic calcium pump blocker Tg plus 1 mM EGTA in Ca^2+^ -free MLocke’s K5 buffer [30]. Then, SOCE was measured after adding 3 mM of CaCl_2_ to the Tg-containing medium. Data acquisition and analysis were carried out using HCImage software after the selection of the cells using the ROI tool of this software. 

### 4.6. Statistics

Statistical analysis was carried out using the Student’s *t*-test, and the results are expressed as the mean ± standard error of the mean (SEM). A significant difference was defined as *p* < 0.05. All results were obtained at least in triplicate, with the number of cells (*n*) indicated for each experimental condition.

## Data Availability

Not applicable.

## Sample Availability

Not available.

## Abbreviations

Aβ	amyloid β peptide
AD	Alzheimer’s disease
APP	amyloid β protein precursor
BACE1	β-site APP-cleaving enzyme 1
C1q	complement component 1q
DMEM	Dulbecco’s modified Eagle’s medium
EGTA	ethyleneglycoltetraacetic acid
ER	endoplasmic reticulum
Fluo3 AM	Fluo-3-pentaacetoxymethyl ester
Fura2 AM	Fura2 acetoxymethyl ester
GAPDH	glyceraldehyde-3-phosphate dehydrogenase
GF	green fluorescence
HEPES	N-[2-hydroxyethyl] piperazine-N′-[2-ethanesulfonic acid]
IL-1α	interleukin-1α
NPA	3-nitropropionic acid
PBS	phosphate-buffered saline
PBST	PBS supplemented with 0.2% 4-(1,1,3,3-tetramethyl butyl) phenyl-polyethylene glycol (Triton X-100^®^)
PMCA	plasma membrane calcium pump
PSEN	presenilin
PVDF	polyvinylidene difluoride
RF	red fluorescence
ROI	region of interest
SDS-PAGE	sodium dodecyl sulfate-polyacrylamide gel electrophoresis
SEM	standard error of the mean
SOCE	store-operated calcium entry
STIM1	stromal interaction molecule 1
Tg	thapsigargin
TNFα	tumor necrosis factor α
